# A deadly taste: linking bitter taste receptors and apoptosis

**DOI:** 10.1007/s10495-025-02091-3

**Published:** 2025-02-20

**Authors:** Zoey A. Miller, Ryan M. Carey, Robert J. Lee

**Affiliations:** 1 Department of Otorhinolaryngology, University of Pennsylvania Perelman School of Medicine, Philadelphia, PA 19104 USA; 2 Pharmacology Graduate Group, University of Pennsylvania Perelman School of Medicine, Philadelphia, PA 19104 USA; 3 Department of Physiology, University of Pennsylvania Perelman School of Medicine, Philadelphia, PA 19104 USA

**Keywords:** Taste family 2 receptors, Extraoral taste receptors, Calcium, Cell death, Mitochondria, Phytochemicals

## Abstract

Humans can perceive five canonical tastes: salty, sour, umami, sweet, and bitter. These tastes are transmitted through the activation of ion channels and receptors. Bitter taste receptors (Taste Family 2 Receptors; T2Rs) are a sub-family of 25 G-protein coupled receptor (GPCR) isoforms that were first identified in type II taste bud cells. T2Rs are activated by a broad array of bitter agonists, which cause an increase in intracellular calcium (Ca^2+^) and a decrease in cyclic adenosine 3’,5’-monophosphate (cAMP). Interestingly, T2Rs are expressed beyond the oral cavity, where they play diverse non-taste roles in cell physiology and disease. Here, we summarize the literature that explores the role of T2Rs in apoptosis. Activation of T2Rs with bitter agonists induces apoptosis in several cancers, the airway epithelia, smooth muscle, and more. In many of these tissues, T2R activation causes mitochondrial Ca^2+^ overload, a main driver of apoptosis. This response may be a result of T2R cellular localization, nuclear Ca^2+^ mobilization and/or a remnant of the established immunological roles of T2Rs in other cell types. T2R-induced apoptosis could be pharmacologically leveraged to treat diseases of altered cellular proliferation. Future work must explore additional extra-oral T2R-expressing tissues for apoptotic responses, develop methods for *in-vivo* studies, and discover high affinity bitter agonists for clinical application.

## Introduction

Humans depend on the five external senses: sight, sound, smell, touch, and taste. Of the five senses, taste perception is vital for both nutrient intake and protecting against the intake of toxic substances [1–3]. Humans can perceive five distinct tastes: sweet, bitter, umami, salty, and sour [4]. Beneficial nutrients, like the sugars and amino acids contributing to sweet and umami tastes, activate pleasant (often termed “hedonic”) tastes [5]. Many toxic plant products activate unpleasant (often termed “aversive”) bitter tastes. Bitter taste is thus thought to have evolved, at least in part, to protect against the ingestion of toxic plant products [6].

Taste perception is transmitted through channels and receptors in taste bud cells located on the tongue [1, 7, 8]. In type I and/or type III cells on the tongue, sour and salty tastes signal through membrane channels permeable to H^+^ and Na^+^ ions, respectively, causing the cells to depolarize and release neurotransmitters to afferent gustatory neurons [9–12]. In contrast, sweet, umami, and bitter tastants activate G-protein coupled receptors (GPCRs) in type II taste bud cells. Activation of these GPCRs triggers intracellular cyclic adenosine 3’, 5’-monophosphate (cAMP) and calcium (Ca^2+^) responses as second-messenger signaling molecules [13–18].

Taste family 2 receptors (Tas2Rs or T2Rs) are the GPCRs responsible for bitter taste transmission [19, 20]. In most humans, there are 25 identified T2R isoforms on the tongue [21, 22]. A 26th isoform was more recently identified in two isolated African populations [23]. Some T2Rs are activated by very specific structures of compounds while others are activated by a broad array of diverse ligands, including compounds like denatonium benzoate, used in products to prevent accidental ingestions, or acyl-homoserine lactones (AHL) used in gram-negative bacterial quorum sensing [24–26]. Fitting with a role for T2Rs in the detection of plant toxins, evidence suggests that the number of T2R isoforms in an animal species correlates with the fraction of plants in the species’ diet [27].

**Fig. 1:**
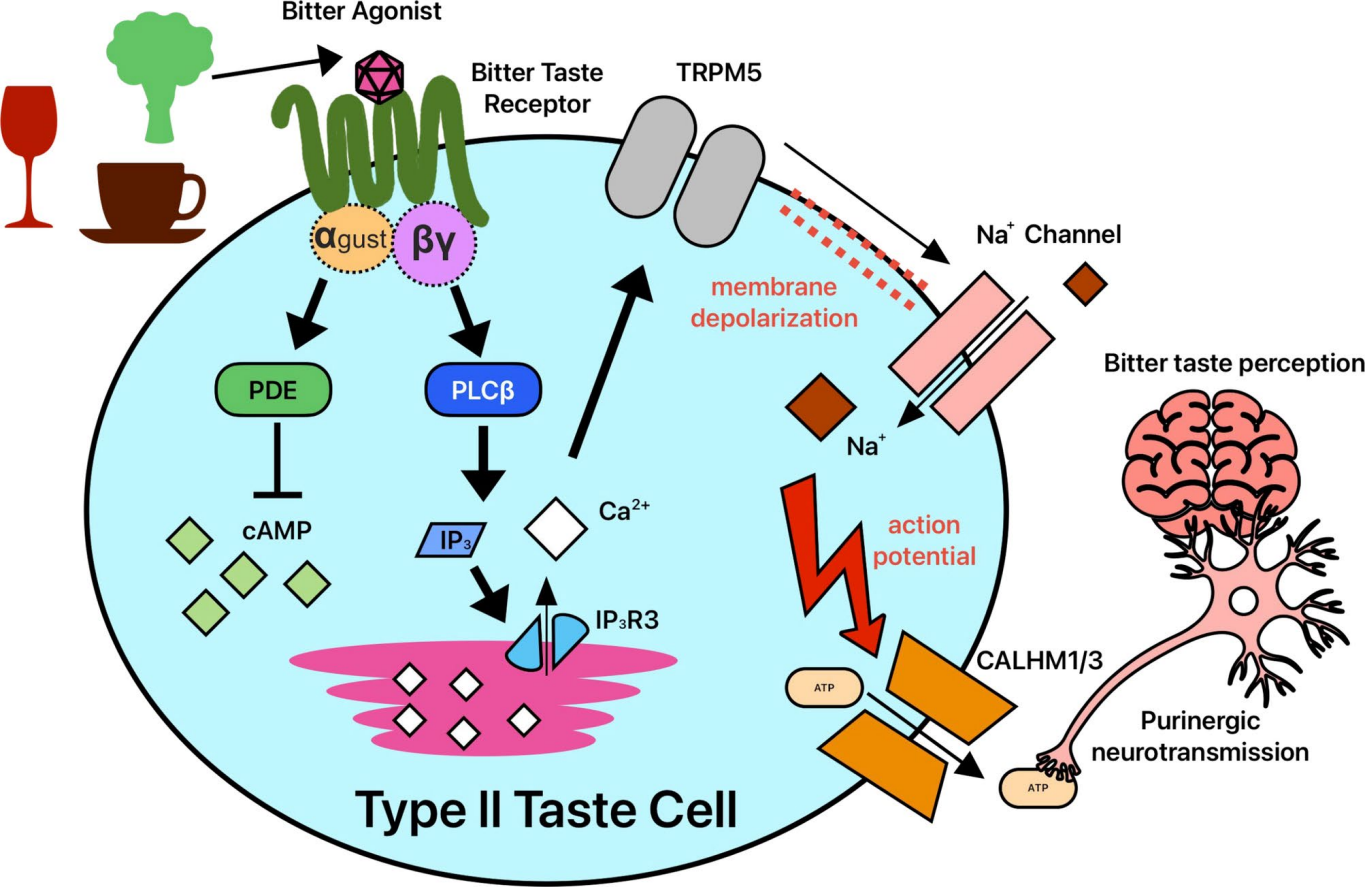
Bitter Taste Signaling Pathway. Canonical GPCR bitter taste receptor (T2R) signaling in type II taste bud cells as described in text

When T2Rs are activated, T2R-coupled G protein isoforms, including Gα_gustducin_, Gβ_1/3_, and Gγ_3/13_ [13, 19, 28–30], disassociate from the receptor and orchestrate cAMP and Ca^2+^ responses in type II taste bud cells. The T2R-evoked Ca^2+^ response activates Na^+^-permeable TRPM5, which depolarizes the cell membrane and causes non-vesicular ATP release through a large conductance ion channel complex containing voltage-dependent CALHM1 and CALHM3 proteins (Fig. 1) [31]. Atypical mitochondria with large tubular cristae [32] located adjacent to the CALHM1/3 complexes [33] produce ATP that likely diffuses directly through the CALHM1/3 pore [34]. The released ATP activates nearby gustatory nerves via purinergic P2X receptors [31]. These signals travel to and are processed by the gustatory cortex in the brain, thus allowing us to perceive taste [35]. Bitter type II cells are associated with aversive gustatory neurons, while sweet and umami type II cells are associated with hedonic gustatory neurons [36].

The vast array of compounds that can activate T2Rs has led to their consideration as a “promiscuous” family of GPCRs [22, 37]. For example, the T2R14 isoform can recognize many diverse structures, including diverse clinically used drugs like diphenhydramine [38] and bitter plant flavonoids [39, 40]. This receptor promiscuity has been attributed to inherent flexibility of the receptor structure, allowing it to conform to structurally diverse agonists [41]. Other T2Rs, like T2R5, appear to bind to fewer structural types of agonists [42, 43]. One factor that influences agonist-receptor specificity is the variation in amino acid sequence, specifically in transmembrane domain 7 [44]. An added level of complication to the T2R family is the ability of nearly every T2R isoform to heterodimerize with nearly every other T2R isoform [45] and possibly other GPCR types [46]. While heterologous over-expression studies have not revealed any obvious consequences of heterodimerization [45], this has not yet been investigated for endogenous T2Rs.

Recently identified CryoEM structures of T2R14 have revealed multiple potential agonist binding sites, one of which may interact with cholesterol [47–49]. While previous studies have shown that membrane cholesterol can modulate T2R14 function [50], this may be more of a direct effect than previously thought if cholesterol acts as a *bona fide* agonist or allosteric modulator of T2R14. These new structural models, combined with the also recently-derived crystal structure of T2R46 bound to its agonist strychnine [51], will likely reveal new insights into structure–function relationships in the T2R family. Such insights are critically needed because our general biochemical and biophysical knowledge of the T2R family lags well behind other more well-studied GPCR families. Beyond a few specific agonists and inhibitors of T2R14 [37, 52], we lack good pharmacological tools to specifically manipulate most of the T2R isoforms. Antagonists in particular are critical for understanding the roles of specific T2R isoforms. Pharmacological tools may also be therapeutically useful, given the emerging role of T2Rs in many processes beyond taste, described in the next section.

Notably, because bitter taste is generally considered to have evolved to protect us from ingesting poisons [6, 27], many of the pharmacological tools we have for activating T2Rs are substances that have off-target, often cytotoxic effects. Many T2R agonists used also come from known pharmaceutical drugs originally used for other mechanisms of action [38]. Results using T2R agonists like lidocaine (Na^+^ channel blocker), colchicine (microtubule destabilizer), and flufenamic acid (cyclooxygenase and Cl^−^ channel inhibitor) must be interpreted with caution and only in light of rigorous knockdown/knockout or other genetic studies. Many studies use multiple, structurally different agonists or antagonists to elucidate receptor function and guard against off-target effects of any single type of agonist. Nonetheless, this underscores a common theme of T2R research, namely a need for both higher affinity and more specific pharmacological tools to more rigorously study these receptors.

### T2R signaling beyond the oral cavity

At least some T2R isoforms are expressed beyond the tongue, termed “extraoral” T2Rs [53, 54]. However, mammals do not perceive taste in these regions. It appears that T2Rs are better thought of as chemosensory GPCRs used in taste but not necessarily specific for the process of taste. Do extraoral T2Rs function and which roles do they play in non-taste tissues? Surprisingly, extraoral T2Rs, both at baseline and with bitter agonist stimulation, can play roles in thyroid function, innate immunity, and cardiac function [55–57]. Furthermore, intracellular Ca^2+^ mobilization—the primary response triggered by T2R activation—plays a critical role in numerous cellular processes. As a result, the influence of T2Rs on cell physiology is only beginning to be understood [58–61].

As occurs in taste cells [62], activated extraoral T2Rs cause an intracellular increase in Ca^2+^ and a decrease in cAMP [15, 25, 63] resulting from G-protein dissociation and signaling. The Gα subunits linked to T2Rs (typically Gα_i,_ Gα_o_, or Gα_gustducin_) typically inhibit cAMP production, either through adenylyl cyclase inhibition (Gα_i_ or Gα_o_) or phosphodiesterase activation (Gα_gustducin_) [28, 64, 65]. The Gβγ subunits induce an intracellular Ca^2+^ response typically via stimulation phospholipase C (PLC), specifically via the PLCβ2 isoform in taste cells [19, 66–68]. Other PLC isoforms may be activated in extraoral tissues. PLC cleaves phosphatidylinositol 4,5-biphosphate (PIP_2_) to generate inositol 1,4,5-triphosphate (IP_3_) [58], which activates endoplasmic reticulum (ER) Ca^2+^ release [69] via the IP_3_ receptor (IP_3_R) [70]. The concentration of Ca^2+^ in the ER is several orders of magnitude higher than the resting ~ 100 nM Ca^2+^ in the cytoplasm, making the ER a major store of intracellular Ca^2+^ that can rapidly be released into the cytoplasm [71, 72]. The IP_3_R is a ubiquitous ER-localized intracellular Ca^2+^ channel expressed in nearly every cell in the body [71, 72], and thus T2Rs are likely poised to activate Ca^2+^ responses in nearly any cell type expressing these receptors. Other Ca^2+^ signaling pathways have been linked to Gα_i_ activation with other GPCRs, including activation of Ca^2+^ permeable TRPC4 and TRPC5 channels [73]. While these TRP channels have not yet been implicated in T2R signaling to our knowledge, they remain potential downstream targets of extraoral T2Rs.

Instead of signaling to gustatory neurons on the taste bud, the extraoral T2R-evoked Ca^2+^ responses likely play diverse roles in human physiology. Ca^2+^ is a dynamic and ubiquitous signaling molecule that is a master regulator of cell physiology [74], as it binds to many proteins directly or can indirectly affect protein function through calmodulin or other downstream effectors. Muscle contraction, exocytosis, gene expression and epigenetics, and many other processes are regulated by IP_3_R induced Ca^2+^ signaling [75] and may thus be regulated by T2Rs.

### T2Rs in innate immunity

Early after their discovery, extraoral T2Rs were identified as distinct players in innate immunity [76]. T2R activation in macrophages stimulates Ca^2+^, nitric oxide (NO), and cyclic-GMP signaling, which drives phagocytosis of bacteria [77]. Taste receptors have also been tied to regulation of neutrophil migration [78, 79]. The role of T2Rs in immunity appears to be linked to their detection of bitter bacterial metabolites, like acyl homoserine lactones (AHLs) [80, 81] and quinolones [25] secreted by gram-negative bacteria like *Pseudomonas aeruginosa*.

In the respiratory tract, T2Rs are expressed in ciliated epithelial cells [56, 82] and solitary chemosensory cells (also known as tuft cells) [56] that regulate local innate defense responses. In nasal cilia, the T2R38 isoform can sense gram-negative AHLs and quinolones and stimulate Ca^2+^-dependent NO production to increase mucociliary clearance and directly kill bacteria [25, 83, 84]. Polymorphisms in the *TAS2R38* gene encoding T2R38 contribute to susceptibility of patients to gram-negative upper respiratory infection [56, 85–87] and/or chronic rhinosinusitis [88–91], play a role in sinus disease severity [92], and contribute to surgical outcomes in patients undergoing functional endoscopic sinus surgery [93]. In cystic fibrosis (CF) patients, *TAS2R38* polymorphisms may also play a role in pulmonary *P. aeruginosa* lung colonization [94]. In fact, the entire T2R to NO signaling pathway is disrupted in CF cells, which may contribute to the intrinsic sensitivity of CF patients to gram-negative respiratory infections [95].

Like T2R38, T2R14 can detect quorum-sensing molecules from cariogenic *Streptococcus mutans* and aid in immune responses in gingival epithelial cells [96]. T2R14 may also modulate bacterial internalization in oral epithelial cells [97]. Beyond immunity, activation of T2Rs in airway smooth muscle causes bronchodilation, yielding a new class of compounds to treat obstructive lung disease [98]. Polymorphisms in T2Rs can affect a variety of physiologic processes and pathologies, including aging, chronic ear infection, rhinosinusitis, thyroid function, and more [55, 99–101]. Naturally occurring polymorphisms in T2Rs that alter contractile function have recently been found in cardiac muscle [97]. It is clear that these receptors serve many purposes in non-gustatory tissues, both in immunity and beyond.

**Fig. 2:**
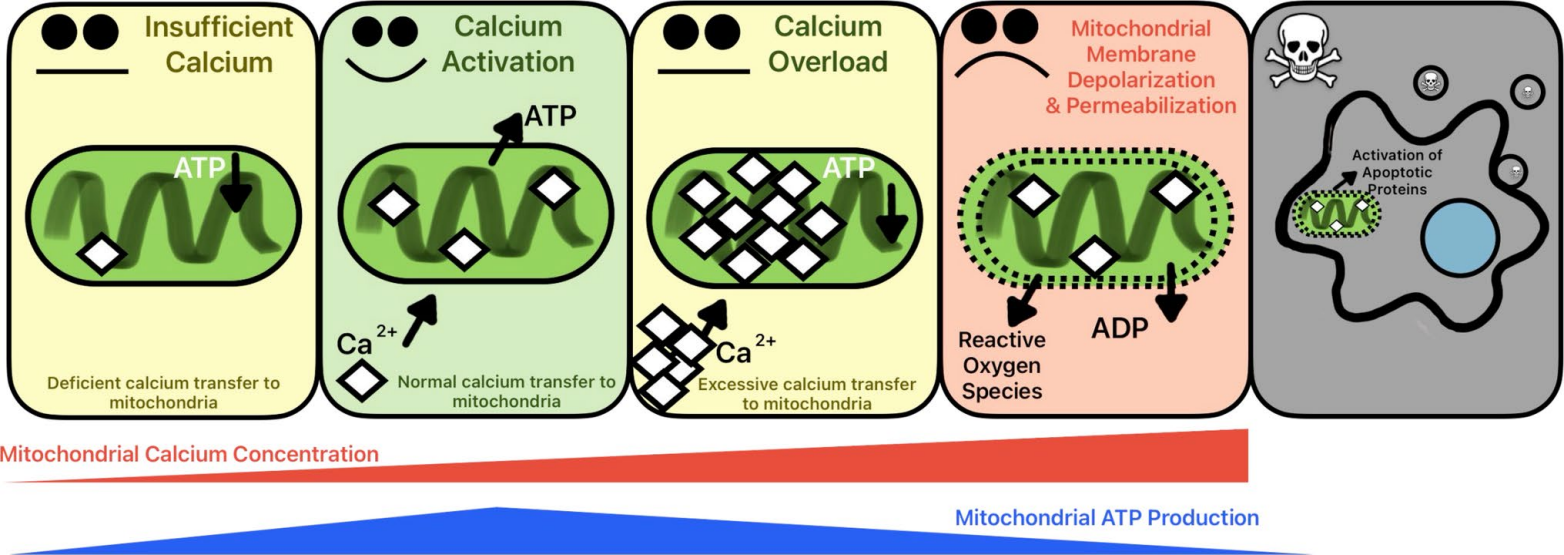
Mitochondrial Ca^2+^ Homeostasis. At moderate levels, mitochondrial Ca^2+^ promotes ATP production [114]. Conversely, mitochondrial ATP production is hindered when the organelle has too much Ca^2+^, known as mitochondrial Ca^2+^ overload [115], or has insufficient Ca^2+^. This disruption causes mitochondrial membrane depolarization and permeabilization, leading to the production of reactive oxygen species [60]. The mitochondrial damage from excessive Ca^2+^ triggers apoptosis [116]

A recently discovered outcome of extraoral T2R activation in some cell types is apoptosis [16, 17, 102]. This is a result of T2R intracellular Ca^2+^ mobilization. IP_3_R-induced Ca^2+^ release elevates the cytoplasmic free Ca^2+^ concentration, but in many cells, this can also result in an influx of Ca^2+^ into other organelles that act as Ca^2+^ buffers, including the mitochondria. Ca^2+^ enters mitochondria via voltage-dependent anion channels (VDACs) in the outer mitochondrial membrane and the mitochondrial Ca^2+^ uniporter complex (MCU) in the inner mitochondrial membrane [103, 104]. As will be described in more detail below, the link between T2R stimulation and mitochondrial Ca^2+^ elevation may be critical to this recently discovered T2R-evoked apoptosis. While transfer of Ca^2+^ into the mitochondria is essential for the maintenance of cellular metabolism [105], excessive Ca^2+^ influx into the mitochondria [106, 107] or impairment of mitochondrial Na^+^/Ca^2+^ exchanger-driven Ca^2+^ efflux [108, 109] can both lead to a condition of having too much Ca^2+^ in the mitochondria, termed mitochondrial Ca^2+^ overload (Fig. 2) [110]. Mitochondrial Ca^2+^ overload has been implicated in the pathogenesis of pancreatitis [111], heart disease [112], and neurodegenerative diseases [113]. As we will discuss below, targeting T2Rs may allow specific induction of mitochondrial Ca^2+^ overload and apoptosis in some types of cancer cells.

T2R-induced apoptosis may be valuable as it could be leveraged against hyper- or hypo-proliferative diseases, including cancer. It also reveals that bitter agonists for T2Rs could be able to be used as therapeutics in disease. Here, we examine the current literature highlighting T2R apoptosis regulation, the underlying mechanisms of this response, and the possible applications of T2R activation with bitter agonists.

### T2R-induced apoptosis in cancer

Cancer is a leading global cause of death [117]. Although the development of targeted, biological, and immune therapies has dramatically improved cancer outcomes, patients continue to experience the severe morbidities associated with treatments and face low survival rates [118]. GPCRs are drivers in cell survival and death and are implicated in cancer [119]. However, only a handful of cancer therapeutics target GPCRs [120]. Several recent studies have asked if extraoral bitter taste GPCRs function in cancer and if they can be pharmacologically leveraged.

**Fig. 3:**
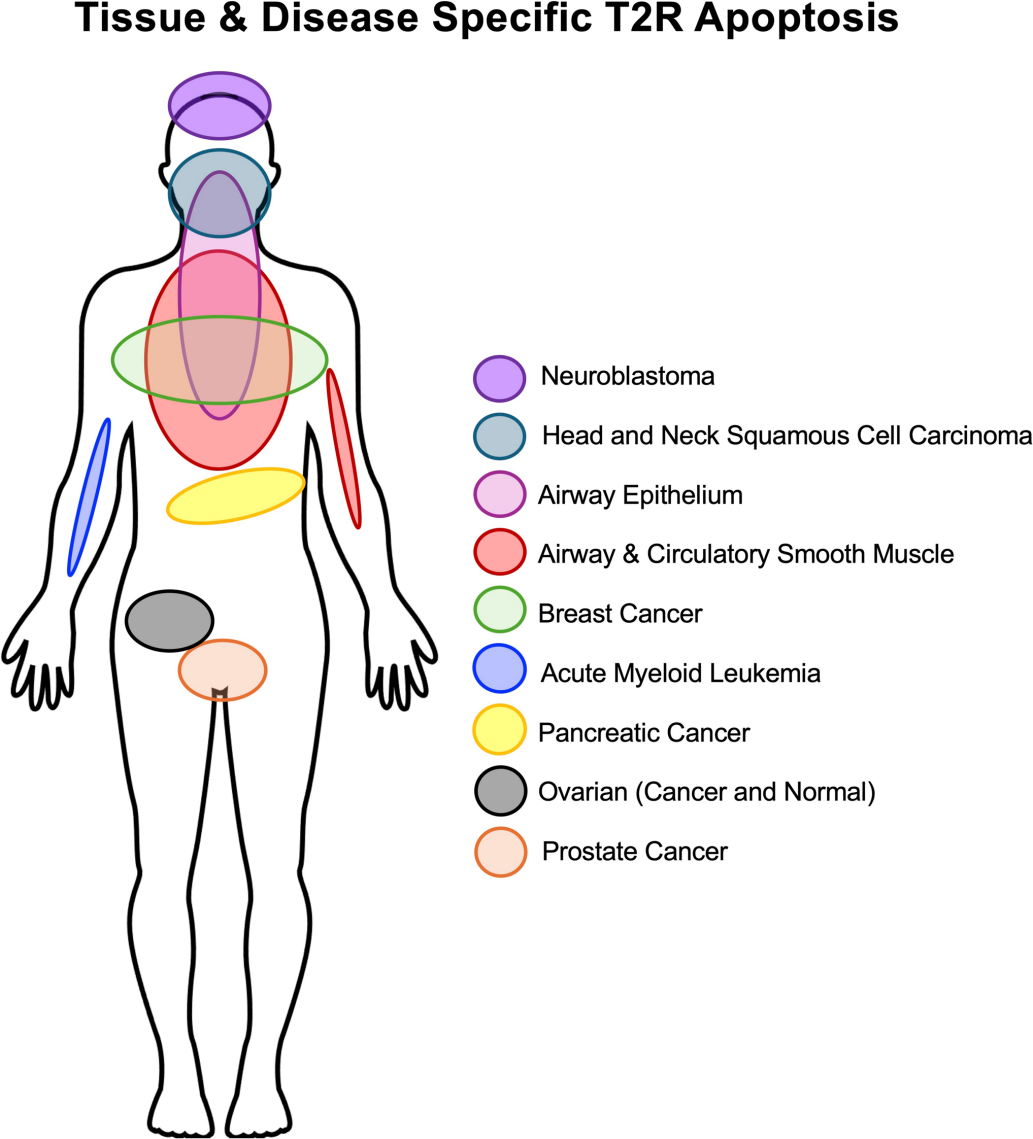
Extraoral T2R Localization. Diagram of the human body indicating documented regions/tissues/cell types in which bitter taste receptor T2R activation induces apoptosis, based on references described in the main text

T2Rs are indeed expressed in several cancers (Fig. 3) [121]. However, certain T2R isoforms have differential expression patterns in cancer vs normal tissue [16]. Analysis of The Cancer Genome Atlas (TCGA) revealed that higher overall *TAS2R* mRNA expression in head and neck squamous cell carcinoma (HNSCC) correlates with prolonged survival over 5 years [16, 102]. Of the 25 T2R isoforms, heightened *TAS2R4* or *TAS2R14* mRNA expression alone is linked to prolonged survival [16, 102]. High *TAS2R14* and *TAS2R10* mRNA expression are also correlated with longer survival in in pancreatic cancers [122, 123]. This may indicate that T2Rs play roles in survival and act as pro-apoptotic receptors at baseline in some cancers.

However, pan cancer TCGA analysis (32 cancer studies, n = 10,528 tumors) revealed that increased T2R expression is sometimes beneficial and sometimes detrimental to cancer survival [124]. For example, increased *TAS2R14* expression improves melanoma and bladder urothelial carcinoma survival but decreases kidney renal clear cell carcinoma and adrenocortical carcinoma survival [124]. T2R signaling likely varies in different tissues and different cancers, possibly due to different G protein coupling or other aspects of the context in which the T2Rs signal. While we don’t yet know the biological basis for these differences, it nonetheless supports an important role for T2Rs in several cancers. T2R signaling may also be altered or modified by mutations in some cancers, which may explain some discrepancies. Mutations in all T2Rs are found in cancer, including those T2Rs highest expressed in cancers (T2R4, 5, 14, 19, 20, and 49) [124]. Analysis of T2R14 alone showed 56 missense mutations and 13 truncating mutations present in ovarian cancer, uterine carcinoma, lung squamous cell carcinoma, lung adenocarcinoma, cutaneous melanoma, HNSCC, and other cancers [124].

**Fig. 4:**
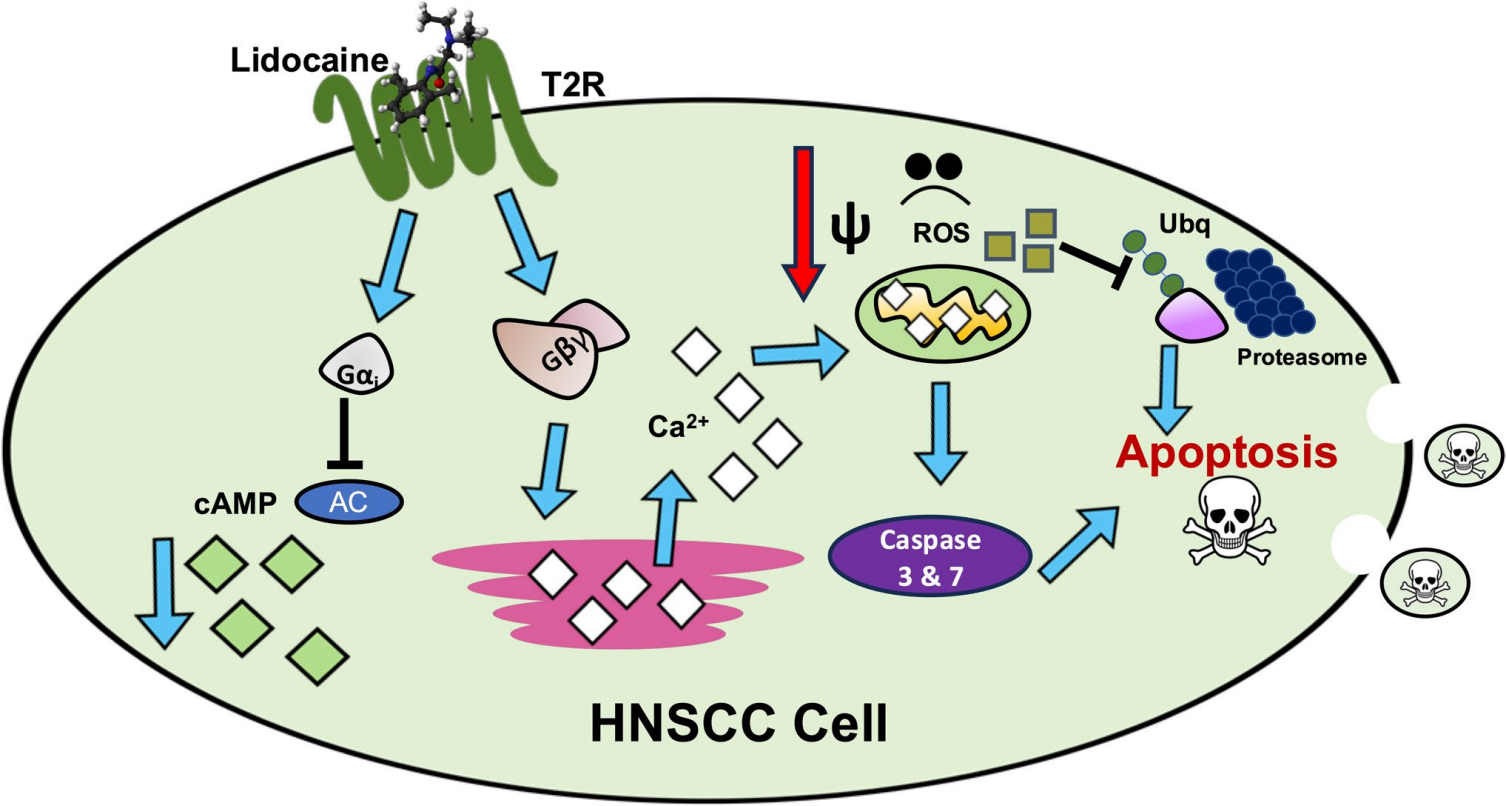
T2R Signaling in HNSCC. As described in the main text, T2R activation with bitter agonists, such as lidocaine, causes a robust Ca^2+^ response. This leads to mitochondrial Ca^2+^ overload, caspase-3 and -7 activation, and production of ROS. ROS inhibits downstream proteasome function, which is an additional player in apoptosis

Activation of T2Rs with bitter agonists induces apoptosis in HNSCC cells (Fig. 4) [16, 102]. Denatonium benzoate, quinine, flufenamic acid, and lidocaine, which collectively activate T2R4, 7, 8, 10, 13, 14, 39, 40, 43, 44, 46, and 47, all kill HNSCC cells. The initial T2R Ca^2+^ response causes mitochondrial Ca^2+^ overload. This stimulates ROS production and downstream inhibition of the proteasome system, which leads to apoptosis. Interestingly, bitter agonists differentially activated Bcl-2 family proteins, which regulate apoptosis [102]. This may indicate differences in signaling mechanisms based on specific T2R isoform activation in HNSCC. Similar apoptotic responses were observed in lung squamous carcinoma cells [102].

Furthermore, while the majority of T2R signaling in taste cells occurs through G_α gustducin,_ we found that T2R isoforms T2R4 and T2R14 may be coupled to different G proteins (G_αq_ vs G_αi_, respectively) [17, 102] further supporting this as a potential explanation for the differential apoptotic signaling in cancer. T2Rs have largely been observed to signal through G_αi_ in extraoral tissues [28], but many GPCRs exhibit promiscuous G protein coupling [125], suggesting T2R signaling in cancer may be strongly influenced by context, including the specific G protein expression within the cells of an individual tumor.

T2Rs also induce apoptosis in breast cancer cells [126]. Quinine and apigenin, which activate T2Rs 4, 7, 10, 14, 39, 40, 43, 44, and 46, decrease cell proliferation and migration, and induce apoptosis through T2R-specific activation. mRNA expression of T2R14 specifically was upregulated in these cells. Similar to T2Rs in HNSCCs, immense Ca^2+^ mobilization in breast cancer leads to activation of Bcl-2 family proteins. Recently, a clinical trial showed that breast cancer patients who received a peritumoral injection of 1% lidocaine before surgery had increased disease-free and overall survival compared with patients without injection [127]. This result may be from direct T2R14 activation with lidocaine as observed with other bitter agonists in breast cancer cells.

In ovarian and prostate cancer cells, noscapine and diphenhydramine, agonists of T2R14 and T2R40, decreased cell viability and induced apoptosis [128]. The mRNA expression of T2R isoforms 1, 4, 10, 14, and 38 were either unchanged or downregulated in prostate and ovarian cancer cells (compared to normal tissue from cell line origin). While heightened T2R expression may be correlated with better cancer patient survival, some cancer cells may downregulate these receptors to escape anti-tumor activity [121].

Some studies are investigating the sensitization of cancer cells to traditional chemotherapeutics with bitter agonists. In pancreatic cancer cells, the bitter agonist caffeine alone did not decrease cell viability. Combining it with chemotherapeutics, gemcitabine or 5-fluorouracil, enhanced these effects [123]. Sub-apoptotic concentrations of lidocaine and denatonium enhanced the effects of a novel GLUT1 inhibitor, BAY-876, in HNSCC, showing the possibility of combinatory therapy with bitter agonists [129].

Similar apoptotic effects with T2R activation have been observed in models of acute myeloid leukemia (AML) and neuroblastoma. Denatonium benzoate altered mitochondrial bioenergetics and induced apoptosis in AML cells, showing that T2R activation is functional in non-solid tumors [130]. T2R10 overexpression alone (not with bitter agonist) hindered the growth of neuroblastoma spheroids and decreased cell migration [131]. Apoptosis, however, was not measured.

It has long been hypothesized that endogenous ligands in the human body may regulate T2R activity. The recently identified CryoEM structures of T2R14 revealed a binding site for cholesterol, suggesting possible modulation of T2Rs by cholesterol levels [47–49]. One study found lower total cholesterol levels in patients who died from several cancers, including pancreatic and breast cancers which express T2Rs [132]. However, effects of cholesterol through T2Rs in malignancy remain speculative. While cholesterol levels could affect T2R14 activity in cancer, another interesting possibility is the modulation of T2Rs by cholesterol-derived steroid hormones. Progesterone was shown to be an agonist of both mouse and human T2R isoforms in heterologous expression studies [22]. Other steroid hormones remain to be tested. T2Rs have been suggested to regulate steroid hormone production in both ovaries [133] and testes [134, 135], and their activation by endogenous steroid hormones may play part of a feedback loop in normal tissues. This may also influence cancer cell biology in these tissues where T2Rs are expressed. Both endogenous hormones as well as exogenous prescribed glucocorticoids have long been known to influence both immunity [136] and the cancer microenvironment [137]. The rapid effects of many steroid hormones on cells has led to the hypothesis that these steroids have both classic nuclear steroid receptors as well as more quickly responding GPCRs [138, 139]. This has been studied with estrogen, which activates both the classic estrogen receptors α and β as well as the G protein-coupled estrogen receptor (GPER) GPR30 [140, 141]. T2R activation by other steroid hormones may underlie some of the “rapid effects” of steroids observed in some cells and tissues. This may also play an important role in influencing cancer cell metabolism or proliferation.

As discussed above, T2Rs can be activated by bacterial metabolites, most notably quinolones and AHLs from gram-negative bacteria [80, 81, 142] but also possibly competence stimulating peptides secreted by cariogenic *Streptococcus mutans* in the oral microbiome [96]. The tumor microenvironment (TME) of many cancers contains bacteria, which also exist in the normal and transient microbiota of skin and mucosal surfaces [143]. Quorum-sensing AHL 3-oxo-C12HSL from *P. aeruginosa*, induces apoptosis in HNSCC cells through T2R14 activation [144]. *P. aeruginosa* can be detected in the oral microbiome, and HNSCC patients often have increased colonization of *P. aeruginosa* [145, 146]. The presence of *P. aeruginosa* in tumors has been correlated with positive prognosis [147]. However, the concentrations of 3-oxo-C12HSL that activate apoptosis in vitro (10-100 µM) are levels typically found in *P. aeruginosa* infections rather than colonization. While biofilm concentrations can reach ~ 500 µM, low level colonization would not be expected to be associated with high µM levels of AHLs. However, 3-oxo-C12HSL may have effects at lower levels when combined with other host or bacterial T2R agonists in the TME. Alternatively, 3-oxo-C12HSL activation of T2R14 may be more important during *P. aeruginosa* infections observed in HNSCC [148] and other cancer patients [149, 150]. As a “proof of principle,” however, the activation of T2Rs by bacterial metabolites like 3-oxo-C12HSL suggests that TME bacteria might produce physiologically relevant ligands for T2Rs that contribute to cancer progression. Further identification of bacterial T2R ligands is needed.

T2Rs offer a largely unexplored and unexploited family of receptors that can induce apoptosis in cancers. Beyond the therapeutic potential of bitter agonists, the upregulation of certain T2Rs in cancer shows promise for targeted treatments. Although T2Rs are low-affinity receptors and bitter agonists are often used in the millimolar range, some cancers, like HNSCCs, allow for localized treatment using injection or mouthwash. This could be clinically relevant, especially with T2R research on lidocaine, which is FDA-approved and used frequently in the HNSCC clinic. Beyond therapeutics, expression and survival data open a new realm of exploring T2Rs in the context of pro-tumor genetic programming. More work is warranted to understand how these receptors function in the tumor microenvironment with endogenous/naturally occurring bitter ligands that are currently unknown.

### T2R-induced apoptosis in airway epithelia

The airway epithelium is a dynamic layer of cells that acts as a barrier to foreign pathogens. This lining begins in the nasal cavity and extends into the far lung [151]. Within the human airway epithelium, T2Rs are expressed in solitary chemosensory cells, ciliated epithelial cells, and chemosensory brush cells [152]. Initially, it was discovered that T2Rs played prominent roles in immunity [153, 154]. When activated, T2Rs in airway epithelial cells secrete antimicrobial peptides into mucus, killing pathogens [76]. Beyond activation, mutations in T2R38 correlated with susceptibility to gram-negative upper respiratory infection, such as chronic rhinosinusitis [56]. These findings provide an opportunity to leverage T2Rs against infection and greater insight into extraoral functions.

**Fig. 5:**
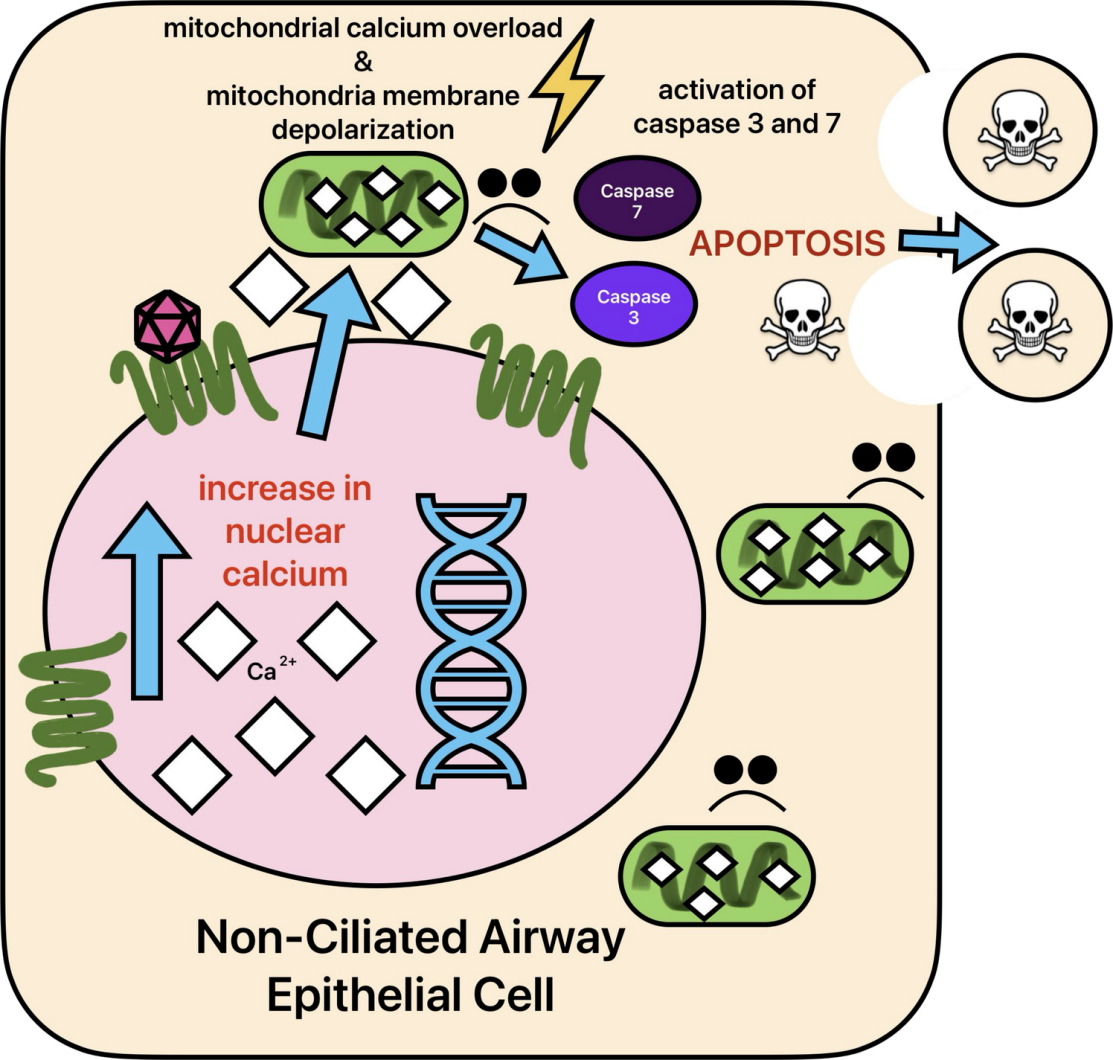
T2R Signaling in Airway Epithelium. Nuclear GPCR T2R signaling causes an increase in intracellular Ca^2+^ and a subsequent overload of Ca^2+^ into the mitochondria, as described in the main text. This causes mitochondrial membrane depolarization and induction of apoptosis in non-ciliated airway epithelial cells

Recently, T2Rs were discovered as regulators of apoptosis in airway epithelial cells (Fig. 5). In non-ciliated epithelial cells, T2R activation induces apoptosis [17]. This is mediated by the specific mobilization of Ca^2+^ into the nucleus and subsequently into the mitochondria. As observed in T2R-induced apoptosis in cancer, mitochondrial Ca^2+^ overload is a main driver of this response. Bitter agonists apigenin, denatonium benzoate, diphenidol, diphenhydramine, flufenamic acid, 3-oxo-C12HSL, parthenolide, phenylthiocarbamine, quinine, and thujone all induced apoptosis, showing that multiple T2R isoforms can trigger this response [17]. Notably, T2R agonists only induced apoptosis in submerged but not differentiated primary human bronchial and sinonasal epithelial cells, showing cell type specificity. In a similar study, denatonium benzoate induced apoptosis via mitochondrial Ca^2+^ overload in airway epithelial cells [155]. Downstream of decreased mitochondrial membrane potential and activation of caspase proteins, Bcl-2 (anti-apoptotic protein) was downregulated and cytochrome c and Smac/DIABLO were released from mitochondria (pro-apoptotic). This uncovers additional underlying apoptotic signaling events from T2R activation (Fig. 5).

The effects of bitter agonists on airway epithelial cell proliferation, but not apoptosis, were also measured [156]. The agonists tested were apigenin, hesperetin, kaempferol, naringenin, quercetin, and naringin. Interestingly, all agonists except naringin decreased proliferation over 48 h. Naringin significantly increased proliferation. While this compound is classified as bitter, its exact cognate T2R isoform(s) are unknown.

T2R activation of apoptosis in airway epithelia may have therapeutic potential. Epithelium hyperplasia is a disease that causes proliferative lesions or cellular remodeling in the airway [157]. In particular, goblet cell or squamous cell metaplasia occurs in diseases like chronic rhinosinusitis, asthma, and cystic fibrosis [158, 159]. Bitter agonists could be used to treat hyperproliferation of the epithelium as the responses may be specific to non-ciliated airway cells [17]. Like the oral and pharyngeal mucosa in HNSCCs, the airway epithelium is accessible to topical therapies and could be treated with bitter agonists, delivered to the nose via saline rinse or spray and delivered to the lung via inhaler or nebulizer.

### T2R-induced apoptosis in smooth muscle

Smooth muscle tissue spans the human body, mainly encompassing the circulatory, digestive, and respiratory systems [160]. These tissues involuntarily contract to mediate digestion, bronchodilation, and more [161]. T2Rs are expressed in smooth muscle cells, and a wide variety of bitter agonists induce an intracellular Ca^2+^ response [98]. Some bitter agonists, like quinine, reduce bronchoconstriction in asthmatic mice [98] This revealed a new non-gustatory role for T2Rs in airway smooth muscle and offers a potential asthma treatment.

T2R activation can induce apoptosis in smooth muscle tissues and cells (Fig. 3). Bitter agonist amarogentin, which activates T2R isoforms 1, 4, 39, 43, 46, 47, and 50, inhibited the proliferation and cell migration and induced apoptosis in vascular smooth muscle cells [162]. This apoptotic response was driven by AMP-activated protein kinase (AMPK) activation in a model of neointimal hyperplasia, using both ligated mouse carotid artery and cultured saphenous vein. Ultimately, amarogentin attenuated the hyperplasia, showing clinical relevance. Other bitter agonists, like chloroquine and quinine, induced cell death in smooth muscle cells, even in the presence of platelet-derived growth factor (PDGF) [163]. T2R activation with these bitter agonists damaged mitochondria, decreased ATP production, and increased ROS. This is similar to the effects observed in cancer and airway epithelial T2R-induced apoptosis. In addition, pharmacological inhibition of autophagy reversed T2R-induced cell death, although it was not elucidated mechanistically how these two processes are connected [163].

The effects of T2R agonists, chloroquine and quinine, were also explored in healthy and asthmatic models of airway smooth muscle (ASM) [164]. Chloroquine and quinine reduced PDGF and epidermal growth factor (EGF)-induced proliferation in normal, asthma, and ASM hyperplasia models. This was caused by downstream inhibition of cell cycle progression gene expression. Cell death was not directly measured; however, there is strong evidence that this may be a result. In a similar study, T2R activation with aloin and papaverine decreased cell proliferation in healthy and asthmatic models [165]. Beyond T2R-activated Ca^2+^ responses, aloin and papaverine caused a quick onset of ERK1/2 (pERK1/2) activation. However, when treated over 24 h, aloin and papaverine downregulated ERK1/2 activation, which was hypothesized to underlie cell proliferation inhibition.

Bitter T2R agonists are often found in traditional, plant-based medicines, including centuries-old herbal remedies [39, 166]. This underlies the ancient phrase used in Chinese medicine, “a good medicine tastes bitter.” Shegan-Mahuang Decoction (SMD) is a Traditional Chinese Medicine (TCM) that has been used for ~ 2000 years to treat cold-like symptoms and contains a plethora of bitter herbs [167]. Administration of SMD to rats improved ASM inflammation and remodeling [168]. SMD upregulated T2R10 and downregulated cell cycle progression genes. This suppressed proliferation and induced apoptosis in ASM. Erythromycin, a specific T2R10 agonist, increased these effects. Qing-Hua Granule, another TCM, may activate T2Rs in mice [169]. Curcumin produces the yellow color of turmeric, also used in many herbal remedies, and contains berberine shown to activate T2Rs in mice [170]. Further exploration of TCM and other traditional medicines could reveal additional bitter compounds and provide more tools to manipulate extraoral T2R signaling in ASM and other tissues.

T2Rs and bitter agonists have the potential to regulate bronchodilation and inhibit hyperplasia or smooth muscle mass growth that is associated with asthma. In addition, those with asthma have a higher risk of developing cancer in their lifetime [171]. Using T2R agonists in smooth muscle airway could effectively treat disease and decrease rates of cancer development due to newly discovered anti-cancer effects.

### T2R-induced apoptosis in other organs/organisms

T2Rs are expressed in cell types and tissues besides cancer cells, airway epithelium, and smooth muscle [172, 173]. Non-malignant ovarian tissue expresses T2R31/44 [174]. Injection of saccharin sodium, which activates T2Rs 43 and 44, inhibits progesterone production and increases T2R44 expression in ovarian tissue of pseudo-pregnant mice. Saccharin sodium also activates p38 and ERK, which results in apoptosis. Contrary to findings in ASM, this activation of ERK, not inactivation, drives an apoptotic phenotype in pseudo pregnant mice [165]. Both states of ERK can induce apoptosis, deeming the protein a “double-edged sword” [175]. Differences in these observations may be due to the specificity of T2R isoforms, tissue type, or dose/length of bitter agonist treatment.

T2Rs are expressed in non-human animals such as yellow-feathered chickens [176]. T2R1, T2R2, T2R7, and other T2R signaling proteins are expressed in jejunal epithelial cells of this species. Dietary administration of denatonium benzoate over 28 or 56 days in the chickens induced apoptosis and upregulated T2R receptors and signaling proteins. In a similar study, long-term dietary administration of denatonium benzoate also induced apoptosis in the heart and kidney of Chinese fast yellow chickens [177]. Denatonium benzoate also increased T2R expression, antioxidant stress, and autophagy in these organs. This study is one of the few that studies long-term exposure to denatonium benzoate in whole organisms. The dietary administration of the compound leaves many questions. The pharmacokinetics of denatonium is unknown. The bioavailability and metabolism of the compound may greatly influence the observed results [178]. More work is warranted to understand the pharmacokinetics of orally administered bitter agonists. 

### Why do T2Rs frequently activate apoptosis in extraoral tissues?

On the tongue, T2R-expressing type II taste cells are bundled together with other taste cells in taste buds, which detect chemical stimuli through taste bud pores opening into the oral mucosa [179, 180]. Type II taste cells appear to be largely immune to bitter-agonist-induced cell death. While one study shows colchicine, a multi-T2R agonist, induces apoptosis in mouse taste buds and epithelial cells [181], most evidence suggests that T2R-induced apoptosis is specific to extraoral tissues. We hypothesize that this difference may be in part due to the localization of these receptors in specific cell types. While taste bud cells typically express T2Rs on the very apical microvillar tips of their cell membranes, which poke out of the taste pore, HNSCC cancer cells, lung cancer cells, and dedifferentiated airway epithelial largely express T2Rs intracellularly [16, 17, 182]. This results in closer proximity of these GPCRs to Ca^2+^-handling organelles, including the ER and mitochondria [183]. Similarly, as described above, well-differentiated airway epithelial cells with T2Rs confined to cilia do not readily die when exposed to T2R agonists. Once de-differentiation occurs and the precise localization of T2Rs is lost, the cells die.

While nuclear membrane and ER-localized GPCRs have been characterized in many cell types [184], the intracellular agonists that activate them are often somewhat mysterious. Intracellular T2R activation could occur via hydrophobic cell-permeant bacterial T2R agonists like lactones or quinolones, produced at high concentrations in biofilms (≥ 500 µM) [142, 185, 186] and kept soluble by bacterial surfactants [187]. In fact, many bitter and sweet taste receptor agonists are cell permeant and are taken up into cells, including into the nucleus [188, 189]. We found that activation of the intracellular T2Rs elicits a strong elevation of nuclear Ca^2+^, which is larger in some cells than the corresponding elevation in cytosolic Ca^2+^ [17]. We hypothesize that the localization and intensity of the nuclear Ca^2+^ responses may elicit intense transfers of Ca^2+^ to the mitochondria that overwhelm the mitochondrial Ca^2+^ buffering capacity and/or the ability of the mitochondrial Na^+^/Ca^2+^ exchanger to lower mitochondrial Ca^2+^, resulting in apoptosis. Other studies have also linked nuclear Ca^2+^ to apoptotic cell death [190, 191]. Similar to our own findings, intracellular T2Rs can also function and regulate Ca^2+^ in keratinocytes [192]. However, intracellular T2Rs in keratinocytes play protective roles against cell death by upregulating ABC transporters. Nonetheless, differences in localization may explain the link between T2R Ca^2+^ and ultimate cell death in extraoral tissues. This difference further shows the potential of bitter agonist therapeutics. Taste bud cells and other well-differentiated cells may remain less affected by T2R apoptosis, making T2R agonists potentially useful therapeutics to target cell death in specific types of cells.

One other reason that T2Rs may be involved in apoptosis is their close association with immunity. Cell death, including apoptosis, is a well-documented component of innate immunity, either as a way for pathogens to invade tissue or host defense mechanism to limit the replication of intracellular pathogens [193]. T2Rs are activated by bacterial metabolites and quinolones [25, 81]. In ciliated airway cells, this activation can stimulate NO production and increase ciliary beat frequency to enhance the mucociliary clearance of bacteria [25, 83, 84]. In cells that don’t produce NO and/or are not ciliated (as such in de-ciliated airway cells), T2Rs may instead induce cell-specific apoptosis as an alternative innate defense to halt infection [17]. Apoptosis is a fundamental and highly effective immune response that can trigger a cascade of downstream responses like immune cell recruitment and inflammation [194]. As this possible immune-associated apoptotic response cannot differentiate between pathogen or non-pathogen related bitter agonists, we hypothesize that this can be exploited therapeutically.

### Conclusion and unmet needs

T2Rs in extraoral tissues play diverse roles in cell physiology and disease. As discussed here and in the literature, recent evidence indicates that these receptors regulate apoptosis upon activation with a variety of bitter agonists in many extraoral tissues (Table 1). The common downstream mechanisms of this activation include nuclear Ca^2+^ elevation, mitochondrial dysfunction due to Ca^2+^ overload and/or modifications in proliferative signaling cascades, such as with ERK1/2 [102, 155, 165]. These findings greatly contribute to our knowledge of extraoral T2R function. It also presents an opportunity to leverage this response against diseases of hyperproliferation or tissue remodeling. More work is warranted to understand why only some extraoral cell types undergo T2R-induced apoptosis.

**Table 1:** Pro-apoptotic bitter agonists

Bitter Agonist	Associated Human T2Rs	Structure	Pre-apoptotic effects	Tissue Type (s)	Ref
N-3-oxo-dodecanoyl-L-Homoserine lactone (3-oxo-C12HSL)	14, 38		Decreased cell viability, depolarization of mitochondria	Airway epithelia	[17]
Aloin	43, 44		Decreased proliferation, inhibition of ERK1/2	Smooth Muscle Airway	[165]
Amarogentin	1, 4, 39, 43, 46, 47, 50		Decreased proliferation and migration, AMPK activation	Vascular smooth muscle	[162]
Apigenin	14, 39		Decreased proliferation and migration, Bcl-2 family protein modulation, depolarization of mitochondria	Breast cancer Airway epithelia	[126, 156, 199]
Chloroquine	3, 7, 10, 14, 39		Damaged mitochondria, decreased ATP, ROS production	Airway Smooth Muscle	[163]
Denatonium Benzoate	4, 8, 10, 13, 39, 43, 46, 47		Decreased viability and proliferation, depolarization of mitochondria, antioxidant stress, proteasome inhibition, autophagy	HNSCC AML Airway epithelia Jejunal epithelia, heart, and kidney of Chinese Yellow Chicken	[16, 17, 130, 155, 176, 177]
Diphenidol	1, 4, 7, 10, 13, 14, 16, 38, 39, 40, 43, 44, 46, 47, 49		Decreased cell viability, depolarization of mitochondria	Airway epithelia	[17]
Diphenhydramine	14, 40		Decreased cell viability, depolarization of mitochondria	Ovarian cancer Prostate cancer Airway epithelia	[17, 128]
Erythromycin	10		Increase TAS2R10 expression, downregulation of cell cycle progression genes, decreased proliferation	Airway smooth muscle	[168]
Flufenamic Acid	14		Decreased cell viability, depolarization of mitochondria	HNSCC Airway epithelia	[16, 17]
Hesperetin	14, 39		Decreased proliferation	Airway epithelia	[156]
Kaempferol	14, 39		Decreased proliferation	Airway epithelia	[156]
Lidocaine	14		Decreased cell viability and proliferation, ROS production, Bcl-2 family protein modulation, proteasome inhibition	HNSCC	[102]
Naringenin	14		Decreased proliferation	Airway epithelia	[156]
Noscapine	14		Decreased cell viability	Ovarian cancer Pancreatic cancer	[128]
Papaverine	7, 10, 14		Decreased proliferation, inhibition of ERK1/2	Smooth Muscle Airway	[165]
Parthenolide	1, 4, 8, 10, 14, 44, 46		Decreased cell viability, depolarization of mitochondria	Airway epithelia	[17]
Phenylthiocarbamide	38		Decreased cell viability, depolarization of mitochondria	Airway epithelia	[17]
Quercetin	14		Decreased proliferation	Airway epithelia	[156]
Quinine	4, 7, 10, 14, 39, 40, 43, 44, 46		Decreased proliferation (PDGF & EGF induced proliferation) and migration, depolarization of mitochondria, ROS production, decreased ATP production	HNSCC Breast cancer Airway epithelia Vascular smooth muscle Airway smooth muscle	[16, 17, 126, 162, 164]
Saccharin Sodium	43, 44		Increased TAS2R44 expression, activation of ERK, NO production	Ovary	[174]
(-)-α-Thujone	10, 14		Decreased cell viability, depolarization of mitochondria	Airway epithelia	[17]

Documented apoptotic bitter agonists listed with associated T2R target(s) [200, 201], agonist structure, pre-apoptotic effects, and location of effect

There are still major gaps within extra-oral T2R research. As previously mentioned, there are only a handful of T2R agonists and inhibitors, many of which activate [22, 38, 173] or inhibit [195, 196] more than one T2R isoform. Recent studies have started identifying more specific antagonists [37], but more work is needed to identify new compounds. Such compounds are vitally needed to elucidate the specific roles and functions that each isoform may play in extra-oral tissues. Recent CryoEM structures of T2R46 [51] and T2R14 [47–49] are expected to aid computational modeling and other screening approaches for antagonist discovery. In addition to their use in research studies, antagonists may have clinical potential. Most studies of T2Rs in cancer cells suggest a pro-apoptotic role, as described above. Nonetheless, there are some cancers, also described above, where increased *TAS2R* expression is correlated with worsened survival. The biological basis of these differences is still unclear, but nonetheless suggest that T2R antagonists might be useful therapeutics in certain cancer contexts. While T2R inhibitors have been used to block effects of T2R agonists in some cancer apoptosis studies [102], to our knowledge the effects of antagonists alone on cancer cells where T2Rs may be contributing to tumor growth have not been studied.

It is important to note that T2Rs are low-affinity receptors that sense bitter compounds in the millimolar range, possibly derived from their contact with high concentrations of bitter compounds on the tongue. Bitter taste on the tongue has been hypothesized to have evolved to protect us from potential plant poisons [6, 27] like strychnine [51] and salicin [197], and thus the off-target effects of known bitter compounds must be taken into account. In some scenarios, localized non-systemic treatments (topical gel or cream, mouthwash, local injections, etc.) with high concentrations of bitter agonists are very feasible, as is the case with lidocaine injections [127]. However, systematic treatments will likely require us to uncover higher affinity bitter agonists through screening of natural compound libraries and/or medicinal chemistry. With the recent solving of cryo-EM T2R structures, antagonists, inhibitors, and high affinity agonists will likely be more easily discovered [48, 49]. However, given that many bitter compounds in research studies must be used at high concentrations and have many off target effects (e.g., strychnine and colchicine [198]), it cannot be stressed enough that these studies must be interpreted with extreme caution unless accompanied by rigorous genetic (e.g., knockdown or knockout) validation.

The effects of extra-oral T2R activation in many disease settings in vivo are still not understood. Due to discrepancies amongst T2R orthologs in humans and mice, in vivo studies are limited outside of taste preference studies. While humans have 25 T2Rs, mice have 35, and many orthologous receptors do not respond to the same agonists [22]. T2R knockout mouse models would have to be interpreted with extreme caution due to ortholog discrepancies. However, the production of human T2R-expressing mouse lines may have some utility for studying whole-organism effects, albeit with confirmation that the human T2Rs are expressed in the same extraoral locations in mice and humans. Nonetheless, because of human and mouse discrepancies, understanding how extraoral T2Rs regulate human physiology is best studied in human culture models. As the state-of-the art human cell culture models progress and can better approximate human tissues (e.g., in the case of differentiated organoid models or organ-on-a-chip models), more translational insights into extraoral T2R biology will likely be revealed.

Finally, literature indicates that extraoral T2R expression and function may be isoform specific, especially in cancer. Advanced sequencing techniques and more rigorous studies using newly discovered inhibitors/antagonists and high affinity agonists could advance our knowledge of the isoform specificity. Advanced sequencing could also reveal extra-oral gene regulation of T2Rs, of which very little is known. The summarized literature and data discussed here are only the beginning of our understanding of extra-oral T2Rs and apoptosis.

## Data Availability

No datasets were generated or analysed during the current study.
